# Rainfall and nitrogen addition have no synergistic effects on steppe composition and production in postgrazing succession

**DOI:** 10.3389/fpls.2025.1635593

**Published:** 2025-09-05

**Authors:** Yue Xin, Qiuxian Huang, Biyao Liu, Zixin Li, Yurong Sun, Min Zhang, Hongwei Yu

**Affiliations:** College of Forestry, Hebei Agricultural University, Baoding, China

**Keywords:** community composition and functions, nitrogen addition, offsetting effects, postgrazing steppe succession, rainfall intensity

## Abstract

Most steppes are experiencing postgrazing succession, coupled with rainfall change and nitrogen (N) deposition. Despite the importance of soil resources in shaping community multifunctionality, little is known about how rainfall increase and N deposition influence steppe composition and production during postgrazing succession. We performed a field factorial experiment, subject to rainfall and N, each with three levels, to understand how simulated rainfall increase and N deposition affect the composition and production of a *Leymus chinensis* steppe. At the low rainfall increase, the dominance of *L. chinensis* increased with increasing N, while aboveground community production remained unchanged along the N gradient. At the high rainfall increase, aboveground community production was enhanced due to low N addition, but this facilitation disappeared in the presence of high N addition, and *L. chinensis* dominance was no longer affected by N. N-induced soil eutrophication but not soil acidification and soil microbes strongly affected steppe composition and production. Our findings suggest that high rainfall increase might weaken the potential of N addition to contribute to steppe composition and production, and also highlight the necessity of investigating interactions among multiple global change drivers.

## 1 Introduction

Plant communities are fundamentally characterized by their composition and production (Gurevitch et al., 2006), which rely tightly on soil resources (Odum and Barrett, 2005; Blair et al., 2014; Fay et al., 2015). Large amounts of soil resources are required to sustain plant communities (Aerts and Chapin, 2000; Schimel and Bennett, 2004; Odum and Barrett, 2005; Swemmer et al., 2007); on the other hand, available soil resources are commonly scarce (Schimel and Bennett, 2004; Elser et al., 2007; Blair et al., 2014; Fay et al., 2015). Thus, it is a basic challenge for plant communities to acquire sufficient soil resources.

The Earth’s ecosystems are experiencing precipitation change and atmospheric nitrogen (N) deposition (Galloway et al., 2004; IPCC, 2023; Zhang et al., 2024). These changes can strongly alter the availability of soil water and N, and thus influence plant community characteristics. For example, increased rainfall can alter plant community composition (Yang et al., 2011; Prevéy and Seastedt, 2014) and enhance community production (Harpole et al., 2007; DeMalach et al., 2017), and decreased soil water can decrease grassland diversity and production (Collins et al., 2014). Increased soil N can impact species richness and community functions (Bobbink et al., 2010; Yang et al., 2011). Water and N addition can yield synergistic effects on plant communities (Harpole et al., 2007; DeMalach et al., 2017), and also generate independent or even antagonistic effects (Zhang et al., 2014; Shen et al., 2022; Peng et al., 2024). Rainfall and N addition also have significant legacy effects (Yu and He, 2017).

Globally, steppes account for a high proportion of grasslands, occupying 30-40% of land surface and covering more terrestrial area than any other single biome type (Blair et al., 2014). Meanwhile, steppes are currently among the most threatened ecosystems due to overgrazing (Blair et al., 2014; Cao et al., 2024). Because of their ecological importance (Blair et al., 2014; Cao et al., 2024), most steppes are now experiencing postgrazing restoration through ceasing grazing (Yu and He, 2019). However, our current knowledge remains quite limited about how enhanced rainfall and N deposition influence postgrazing succession (Yu and He, 2019).

Our previous work focused on the impacts of rainfall and N addition on species turnover (i.e., species colonization and extinction) during postgrazing steppe succession (Yu and He, 2019). We found that the increases in rainfall and N enhanced species losses and gains at the same time and that the rates of species losses and gains were equal. Here we reported how rainfall and N addition influenced steppe composition and production during postgrazing succession. We proposed the following hypotheses: (1) rainfall increase can amplify the potential of N addition to impact steppe composition and production; (2) if so, this effect might be greater at high rainfall increases than at low rainfall increases. To test these hypotheses, we conducted an experiment with a steppe, which was subject to nine different combinations consisting of rainfall and N. We analyzed experimental data using principal component analysis, linear mixed-effects models, and structural equation modeling simultaneously, providing possible mechanisms explaining how rainfall and N addition alter steppe characteristics via soil acidification, soil eutrophication and soil microbes because previous studies have overlooked the relevant processes determining the effects of rainfall and N.

## 2 Materials and methods

### 2.1 Experimental site and designs

This experiment was carried out in a semiarid steppe (49.852° N, 120.348° E, 615 m a.s.l.) in Inner Mongolia, China, which had been severely degraded because of chronic overgrazing. The soil in the study area is categorized as sandy loam. *Leymus chinenesis* (C_3_ plant) and *Cleistogenes squarrosa* (C_4_ plant) were the dominant plant species in the study site before experiment manipulation. See our paper (Yu and He, 2019) for details about study site and plant species at the beginning of the experiment. During the experimental period (2009-2013), the growing season rainfall (327 mm) was greater than the long-term counterpart (294 mm, 1981-2010).

We conducted a field factorial experiment with rainfall and nitrogen treatments designed according to projections by Galloway et al. (2004) and IPCC (2023). Each factor had three levels, yielding a total of nine treatment combinations. Three N levels were: no N addition (N0); an addition of 5 g N m^-2^ yr^-1^ (N5); an addition of 10 g N m^-2^ yr^-1^ (N10). Three rainfall levels were: ambient rainfall (R0); a 14% increase in rainfall amount (R14); a 28% increase in rainfall amount (R28). The full-factorial experimental design was replicated in eight blocks at the study site. Within each block, we constructed nine 2 m × 2 m plots, with 3 m wide aisles separating adjacent plots. These plots within each block were then randomly assigned to the nine different rainfall and N treatment combinations. To achieve a 14% or 28% increase in precipitation amount, one or two rainfall collectors were installed adjacent to the designated plots to simulate changes in precipitation. Each collector was made of iron panels with a basal area of 0.56 m², mounted on a 0.3 m tall timber frame, and connected to the plots via drainage pipes. These pipes were evenly distributed within each plot to ensure uniform water delivery. In parallel, nitrogen was added in the form of NH_4_NO_3_, in dry form, at the beginning of each growing season (i.e., early June). The precipitation and nitrogen addition treatments were continuously implemented from 2009 to 2013. See our paper (Yu and He, 2017, 2019) for detailed information regarding the experimental plots and treatments of rainfall and N.

### 2.2 Measurements of plant community and soil properties

In August 2013, a 1.0 × 1.0 m quadrat was positioned at the center of each plot, and community composition was quantified by recording plant species in the 100 0.1 × 0.1 m squares making up a quadrat. We categorized all plant species into two different functional groups (i.e., C_3_ versus C_4_ plants), and calculated the ratio of C_3_ plant biomass to C_4_ plant biomass to indicate community composition. The aboveground biomass was harvested near the soil surface by clipping within two randomly placed 25 × 25 cm frames in each plot. Meanwhile, we sampled roots (including rhizomes) by taking 25 cm long × 25 cm wide × 15 cm deep soil cores from each clipped area and washed them by hand. The harvested shoots and roots were dried at 75°C for 48 h and weighed.

During plant harvest, soil pH was measured *in situ* using a portable pH meter, and soil samples were randomly collected at a depth of 0-10 cm from each experimental plot. After sieving through a 2 mm mesh to remove debris, each soil sample was divided into three subsamples. The first subsample was stored at 4 °C for the determination of available phosphorus, and ammonium nitrogen (NH_4_^+^-N) and nitrate nitrogen (NO_3_^-^–N); the second subsample was air-dried for the measurement of total carbon, total nitrogen and total phosphorus; the third subsample was frozen at -80 °C for soil microbial community analysis. Phospholipid fatty acid (PLFA) profiling was used to characterize microbial communities, which were classified into bacteria, actinomycetes, fungi, arbuscular mycorrhizal fungi, and protozoa. The PLFA biomarkers used for microbial group identification are listed in **Supplementary Table S1**. Moreover, the fungi/bacteria ratio was calculated to assess changes in microbial community structure.

### 2.3 Data analyses

We measured one soil acidification variable (i.e., soil pH), six soil nutrient variables and six soil microbial variables in this study. To reduce the dimensionality and collinearity of soil characteristics, principal component analyses (PCA) were conducted on the variables related to soil nutrients (PCA_N_) and soil microbes (PCA_M_) separately. The scores of first and second axes of resulting PCA results were used as representative variables and used for the following analyzes.

We first performed linear mixed-effects models to test the effects of N addition, increased rainfall, and their interactions on soil and community characteristics, with block treated as a random term. For variables with significant main or interaction effects, pairwise comparisons were conducted to examine differences among treatment levels or combinations. Then, spearman correlations were performed to explore the associations between plant community characteristics and soil representative variables. In order to improve normality, the ratio of C_3_ plants to C_4_ plants and aboveground biomass were log-transformed when necessary. To figure out how N addition and increased rainfall influenced steppe composition and production, we performed structural equation modeling (SEM) to evaluate the relative importance of soil acidification, soil nutrients and soil microbes induced by N addition and increased rainfall on the ratio of C_3_ plants to C_4_ plant species, and biomass. We assume that N addition and increased rainfall influence steppe composition and production via affecting soil acidification, soil nutrients and soil microbes. The SEM analyzes were performed with the maximum likelihood estimation method. All analyzes were performed with vegan (Oksanen et al., 2022), lme4 (Bates et al., 2015), car (Fox and Weisberg, 2019), emmeans (Lenth, 2023), lavaan (Rosseel, 2012) and ggcor (Huang et al., 2020) packages using R4.3.1 (R Core Team, 2023).

## 3 Results

### 3.1 Effects of rainfall and N addition on steppe performance

**Figure 1:**
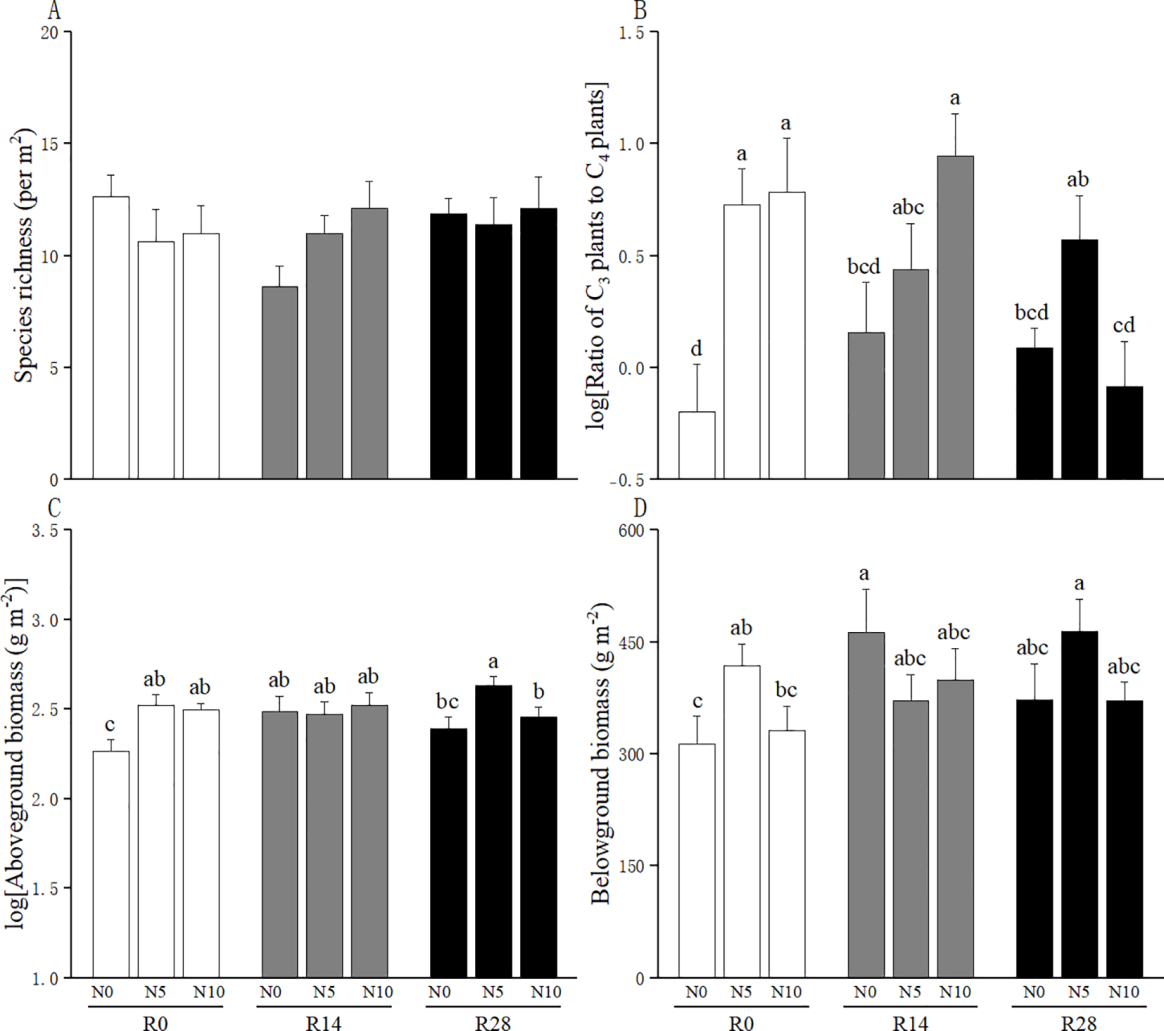
Effects of increased rainfall and N addition on **(A)** species richness, **(B)** the ratio of C_3_ plants to C_4_ plants, **(C)** aboveground biomass, and **(D)** belowground biomass of plant communities. The data are expressed as means + 1 SE. Different lowercase letters indicate significant difference among treatment combinations. N0: no N addition; N5: an addition of 5 g N m^-2^ yr^-1^; N10: an addition of 10 g N m^-2^ yr^-1^. R0: no rainfall addition; R14: a 14% increase in rainfall amount over the ambient rainfall; R28: a 28% increase in rainfall amount over the ambient rainfall.

*Leymus chinensis* accounted for 77% of C_3_ plant biomass and *C. squarrosa* accounted for 99% of C_4_ plant biomass. The linear mixed-effects models results showed that increased rainfall did not alter the species richness and the ratio of C_3_ plant species to C_4_ plant species (**Table 1**; **Figures 1A, B**); N addition increased the ratio of C_3_ plant species to C_4_ plant species, but this N-added effect disappeared at higher rainfall increases (**Figure 1B**). The aboveground biomass was enhanced by N addition rather than increased rainfall; importantly, this facilitation of N addition was substantially offset by higher rainfall increases (**Table 1**; **Figure 1C**). The belowground biomass was unaffected by increased rainfall and N addition, but significantly influenced by their interaction (**Table 1**; **Figure 1D**).

**Table 1:** The effects of rainfall increase, N addition, and their interactions on soil representative variables and community properties.

	Rainfall increase (R)		Nitrogen addition (N)		R × N	
Variable	*χ^2^*	*P*	*χ^2^*	*P*	*χ^2^*	*P*
Soil representative variables						
Microeukaryotes	**6.951**	**0.031**	**11.51**	**0.003**	5.704	0.222
Prokaryotes	0.938	0.626	**11.38**	**0.003**	4.333	0.263
Total nutrients	4.683	0.096	0.731	0.694	**9.763**	**0.045**
Available nutrients	2.309	0.315	**30.57**	**<0.001**	2.772	0.597
Community properties						
Species richness	1.968	0.374	0.914	0.633	6.748	0.150
C_3_/C_4_ ratio	4.637	0.098	**16.23**	**<0.001**	**15.00**	**0.005**
Aboveground biomass	2.452	0.293	**12.07**	**0.002**	**9.744**	**0.045**
Belowground biomass	4.364	0.113	3.104	0.211	**9.638**	**0.047**

Values of *P* ≤ 0.05 are in bold.

### 3.2 Effects of rainfall and N addition on soil properties

Increased rainfall did not alter soil properties except for soil fungi and arbuscular mycorrhizal fungi (**Supplementary Table S2**; **Supplementary Figure S1**). Soil fungi and arbuscular mycorrhizal fungi were more abundant at high rainfall increases than at low rainfall increases. Unlike rainfall enrichment, N addition influenced some soil properties (**Supplementary Table S2**; **Supplementary Figure S2**). For example, soil fungi decreased along the N gradient, N addition increased NH_4_^+^-N and NO_3_^-^–N, and N addition also decreased soil pH (soil acidification) (**Supplementary Figure S2**, **S3**). Additionally, increased rainfall and N addition interacted to influence soil total carbon (**Supplementary Table S2**).

**Figure 2:**
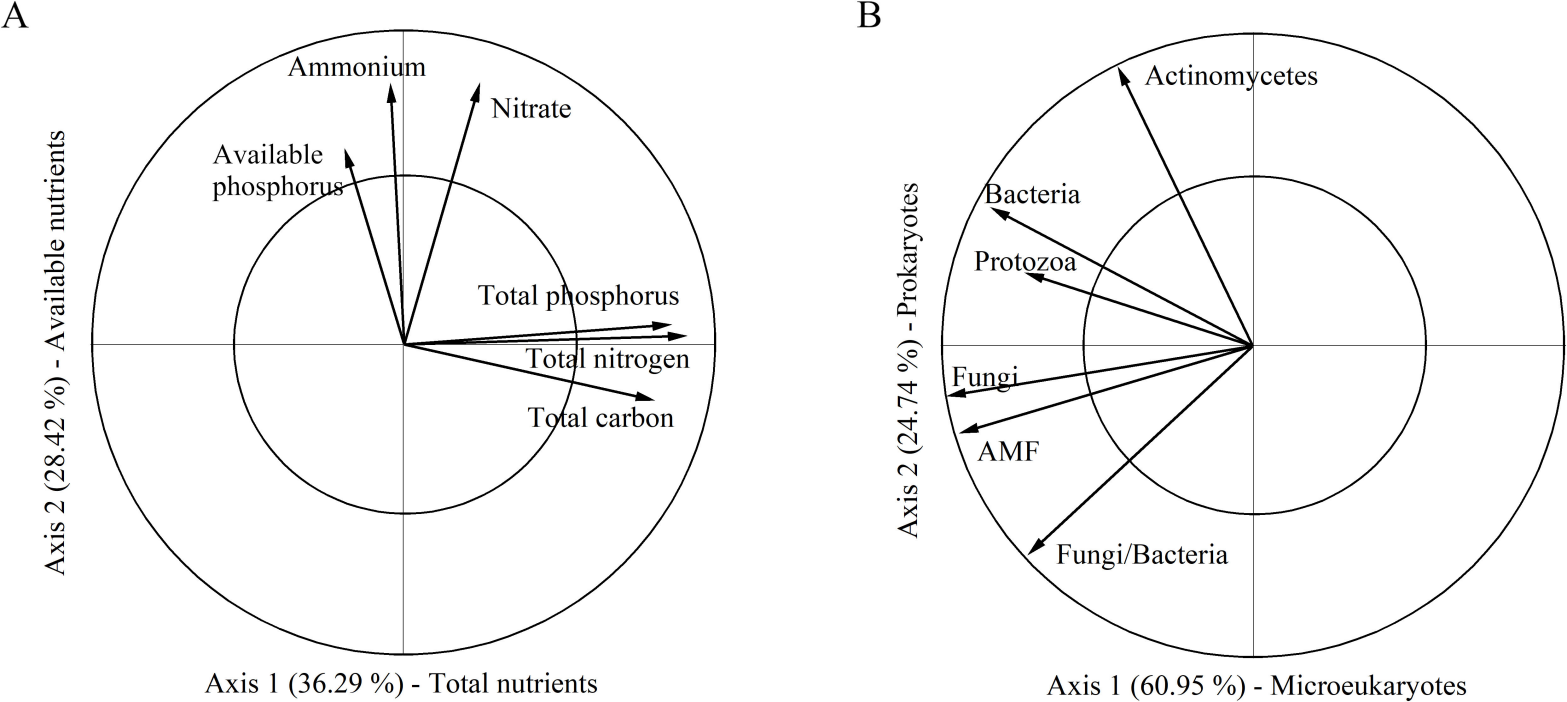
Principal component analysis (PCA) for variables on soil nutrients (PCA_N_) and soil microbes (PCA_M_). **(A)** The first and second axes in the PCA_N_ were categorized as composite measurements of “Total nutrients” and “Available nutrients”, respectively. **(B)** The first and second axes in the PCA_M_ were categorized as composite measurements of “Microeukaryotes” and “Prokaryotes”, respectively.

**Figure 3:**
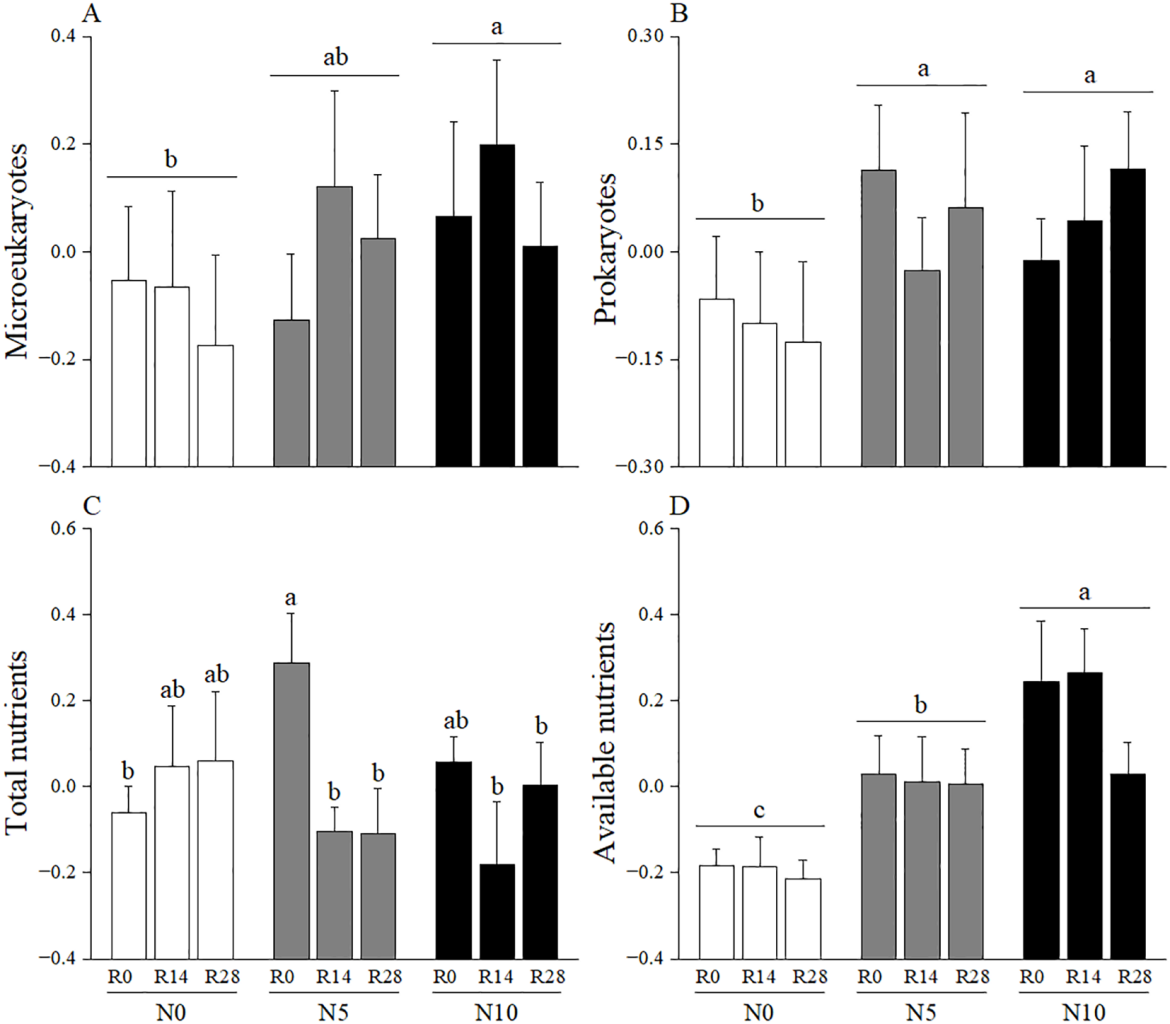
Effects of increased rainfall and N addition on soil representative PCA variables of **(A)** Microeukaryotes, **(B)** Prokaryotes, **(C)** Total nutrients and **(D)** Available nutrients. The data are expressed as means + 1 SE. Treatment abbreviations are from **Figure 1**. Different lowercase letters indicate significant difference among N treatment levels or combinations.

According to the PCA_N_ analysis, the first and second axes explained 36.29% and 28.42% of the variation in soil nutrient, respectively (**Figure 2A**). The first axis was positively correlated with total carbon, total nitrogen, and total phosphorus and thus was categorized as composite measurements of “total nutrients”. The second axis was categorized as measurements of “available nutrients” because it had a high relevance with available nitrogen and phosphorus. A greater value on the first and second axis indicated a high degree of soil eutrophication. In the PCA_M_ analysis, the first two axes explained 60.95% and 24.74% of the variation in soil microbes, respectively (**Figure 2B**). The first axis was categorized as measurements of “microeukaryotes” as it most relevant to fungi, arbuscular mycorrhizal fungi and protozoa. The second axis reflected bacteria, actinomycetes and the trade-off between fungi and bacteria, thus was designated as measurements of “prokaryotes”. In these cases, a greater value on the first and second axes corresponds to a higher microbial biomass in microeukaryotes (first axis) and prokaryotes (second axis). The linear mixed-effects models results showed that increased rainfall significantly affected soil microeukaryotes, while it had no effect on other soil representative variables (**Table 1**; **Figure 3**). Adding nitrogen, regardless of N5 or N10, significantly enhanced soil prokaryotes compared to N0 (**Table 1**; **Figure 3B**). Furthermore, both soil available nutrients and microeukaryotes significantly increased with the increasing nitrogen application rate (**Table 1**; **Figures 3A, D**). In addition, increased rainfall and nitrogen addition interactively affected soil total nutrients (**Table 1**; **Figure 3C**).

### 3.3 Correlations between steppe performance and soil representative variables

**Figure 4:**
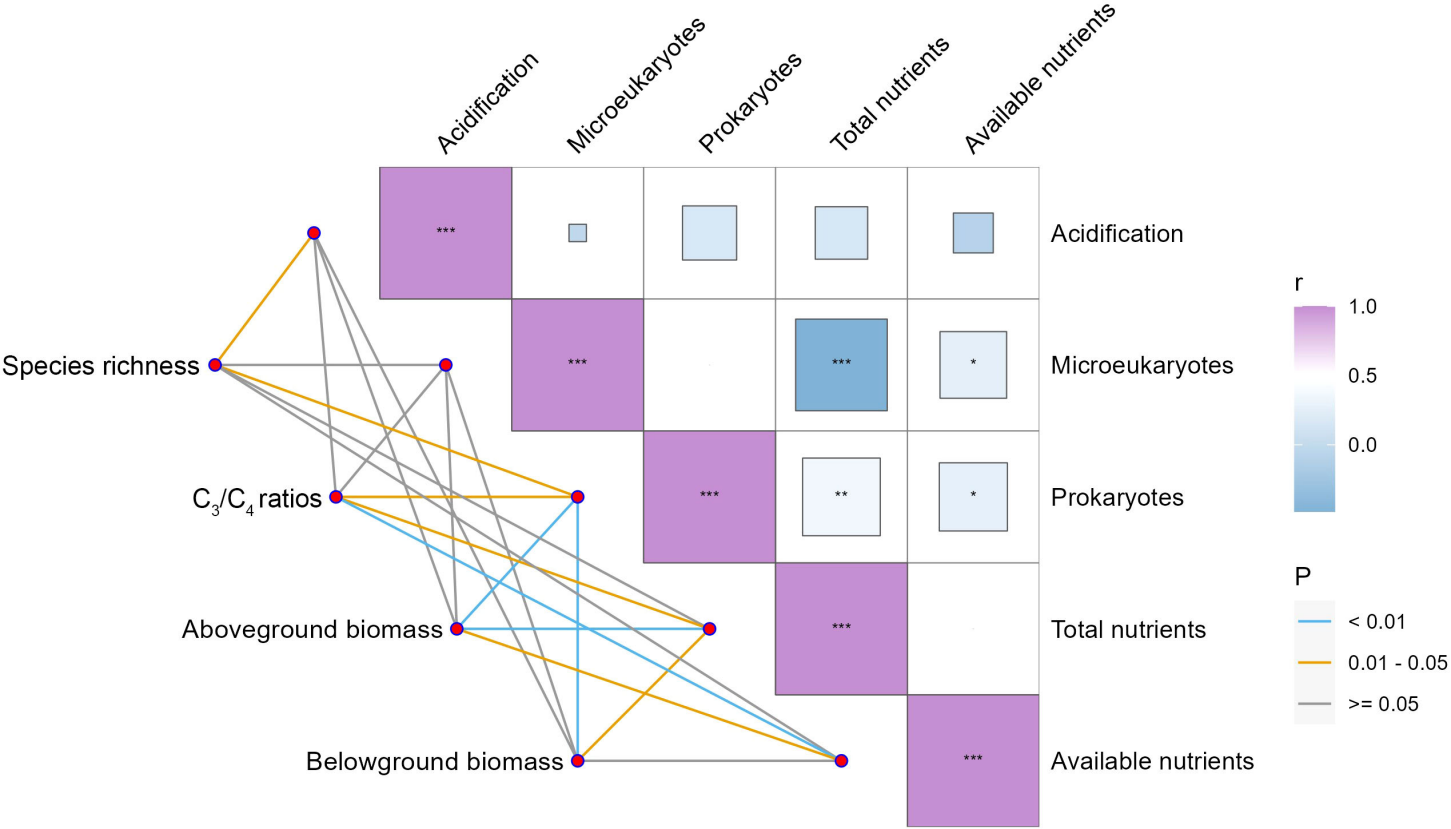
Spearman correlations between steppe performance and soil representative variables. ^*^ *P* < 0.05, ^**^ *P* < 0.01, ^***^ *P* < 0.001.

Species composition and community biomass showed different associations with soil representative variables (**Figure 4**). Soil acidification, as indicated by soil pH, was negatively and significantly correlated with species richness, but no significant associations were detected with other steppe performance indicators (**Figure 4**). Among soil microbes, soil with higher prokaryotes rather than microeukaryotes tended to enhance the ratio of C_3_ plant species to C_4_ plant species and community biomass but decreased species richness (**Figure 4**). Higher soil eutrophication (i.e., total and available nutrients) exhibited significantly positive correlations with community biomass and the ratio of C_3_ plant species to C_4_ plant species but showed no linkage with species richness (**Figure 4**).

### 3.4 Pathways linking field manipulations and steppe performance

**Figure 5:**
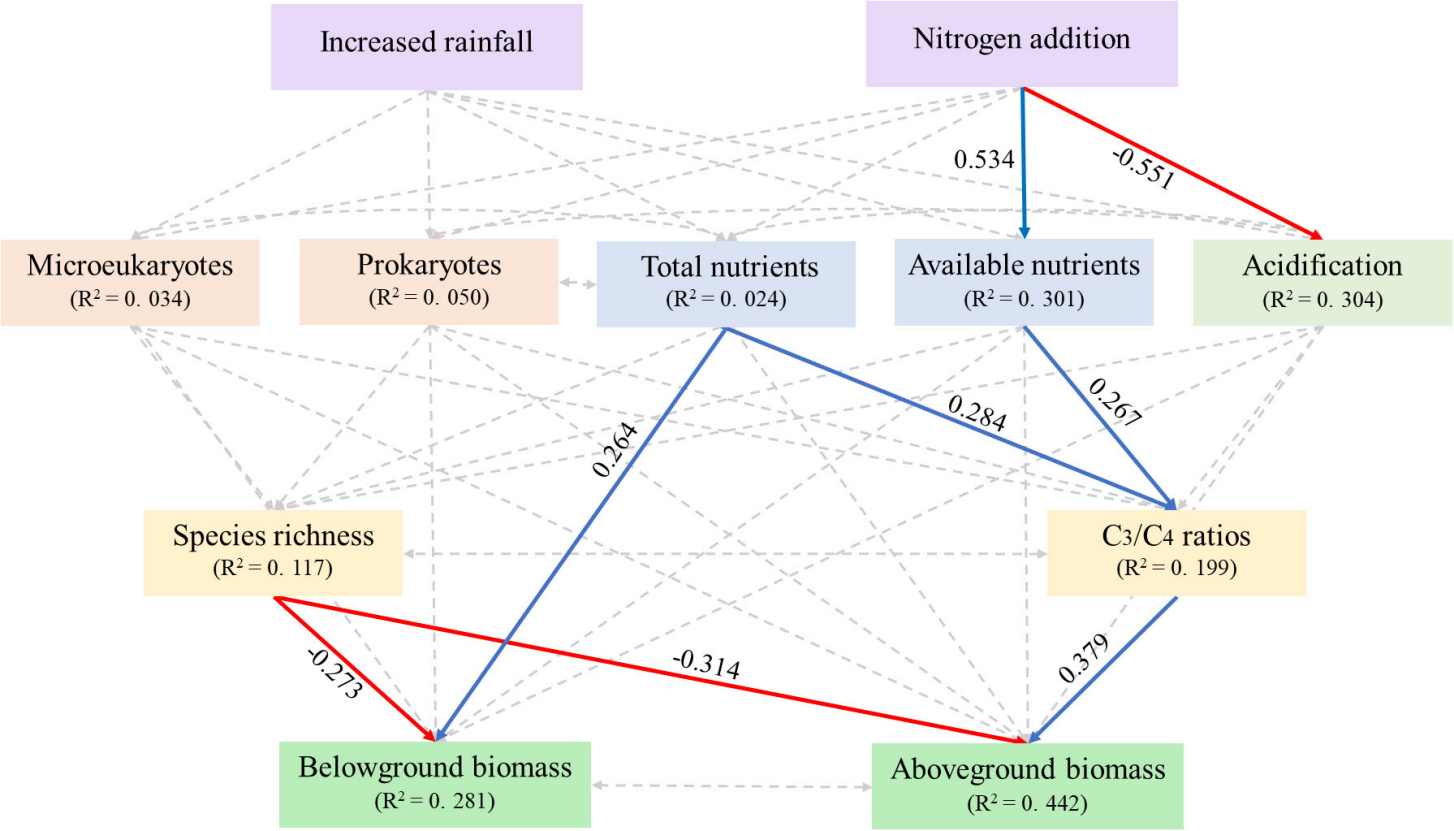
Structural equation modeling examining the effects of increased rainfall and nitrogen addition on plant community composition and production via influencing soil representative variables. Solid red and blue indicate significantly negative and positive effects (*P* < 0.05); the non-significant pathways are shown in grey dot lines (*P* > 0.05). Numbers associated with pathways between variables represent standardized path coefficients. Line width is proportional to the strength of the relationship. χ^2^ = 20.483, *P* = 0.084.

Increased rainfall had no significant effects on steppe composition via affecting soil acidification, eutrophication, and microbes (**Figure 5**). Nitrogen addition increased the abundance of C_3_ species indirectly by increasing soil available nutrient availability (**Figure 5**). Although N addition decreased soil pH (i.e., inducing soil acidification), these alterations had no effects on steppe composition (**Figure 5**). Additionally, soil total nutrients positively influenced the ratio of C_3_ plant species to C_4_ plant species (**Figure 5**).

While the C_3_/C_4_ ratios exhibited a significantly positive relationship with aboveground biomass, species richness was negatively and significantly contributed to both aboveground and belowground biomass (**Figure 5**). The effects of increased rainfall on plant biomass were similar to those on species composition, as no significant pathways were observed in the SEM analysis (**Figure 5**). Nitrogen exhibited a positive effect on aboveground biomass by influencing available nutrients and the C_3_/C_4_ ratios; however, no significant pathways were observed on belowground biomass by influencing soil representative variables and species composition (**Figure 5**). In addition, soil total nutrients were positively correlated with belowground biomass (**Figure 5**).

## 4 Discussion

This work highlighted the influences of simulated rainfall increase and N deposition on steppe composition and production during postgrazing succession. In our study region, the climax plant community is *L. chinensis* communities and *C. squarrosa* is a subordinate species in natural vegetation (IGIMVCAS, 1985). When *L. chinensis* communities degraded due to overgrazing, *L. chinensis* shifted from a dominant species to a subordinate species whereas *C. squarrosa* shifted from a subordinate species to a dominant species (Yu and He, 2019). At the scale of our experimental plots, *L. chinensis* and *C. squarrosa* accounted for majority of C_3_ plant biomass (77%) and C_4_ plant biomass (99%), respectively. Thus, the change in C_3_/C_4_ ratios primarily indicated the shift between *L. chinensis* and *C. squarrosa*. Here, projected rainfall increases had no significant effects on the composition and primary production of a steppe; a N5 or N10 increased C_3_/C_4_ ratios and aboveground primary production (**Figure 1**). These findings suggest that precipitation increase might not strongly influence steppes but simulated N deposition could improve steppes through shifting local steppes from *C. squarrosa* communities to *L. chinensis* communities and increasing their production. As an important global change driver, N deposition has profound impacts on plant community composition, productivity, and biogeochemical cycling of grasslands (Kimmel et al., 2020; Ke et al., 2023; Yuan et al., 2023). However, its ecological efficacy may depend not only on the amount of nitrogen added, but also on the timing of its application (Zhang et al., 2021a; Song et al., 2023). In our study, nitrogen was applied at the beginning of the growing season, aligning with the period of peak plant nutrient demand. This synchrony likely enhanced nitrogen uptake by dominant species such as *L. chinensis*, while reducing potential losses via microbial immobilization. Such timing may partly explain why nitrogen addition effectively promoted C_3_ dominance and aboveground productivity during postgrazing succession.

A key finding of our study was that different strengths of rainfall increases had contrasting impacts on the effects of N addition on steppes. More specifically, at the low rainfall increase, C_3_/C_4_ plant ratio gradually increased with N levels; in contrast, at the high rainfall increase, low-level N addition substantially increased biomass production compared with the controls, but this facilitation disappeared when high-level N was supplied (**Figure 1**). Thus, our findings suggest that high rainfall increase might weaken the potential of high-level N to improve steppes, although they are inconsistent with previous findings (Yang et al., 2011; Ma et al., 2020; Ren et al., 2021).

We propose several possibilities that might explain the above-mentioned findings. Intermediate rainfall events are responsible for most of the rainfall effects for steppes (Sala and Lauenroth, 1982; Lauenroth and Sala, 1992). In our study region, precipitation mainly falls in the form of heavy rainfall events during growing seasons. Large rainfall exceeds the field capacity of soil and causes soil erosion and leaching (Kong et al., 2013; Brown and Collins, 2024), thereby decreasing retention of soil nutrients and influencing N availability in the plant root zone. Second, water pulses can result in a higher N mineralization rate and N mobility (Wang et al., 2006; Lü and Han, 2010; Peng et al., 2024), and N addition can decrease soil water availability by increasing plant photosynthetic rate and transpiration (Harpole et al., 2007; Yang et al., 2011). Third, there are counteractive effects among different ecological processes, which could alter interspecific relationships. Although we did not directly measure the availability of water in the soil in this study, it is important to acknowledge its potential role in mediating the effects of rainfall and nitrogen on steppe ecosystems. Our findings might provide insights into the importance of rainfall increase and N deposition in regulating steppes. For example, increased rainfall may play a weak role in improving steppes, particularly in those areas where heavy rainfall events are frequent in growing seasons. Furthermore, increasing rainfall can offset the positive effects of N on steppes.

We also observed that the importance of soil acidification and eutrophication, which were induced by N addition, differed dramatically. We found that soil eutrophication but not soil acidification strongly affected steppe composition and production (**Figure 5**). These findings disagree with the global synthesis by Bobbink et al. (2010). Interestingly, N-induced soil acidification did not decrease plant species richness (**Figure 4**). Species loss is commonly viewed to be linked to soil acidification, soil eutrophication, and competitive exclusion (Stevens et al., 2010; Kimmel et al., 2020; Ke et al., 2023). However, these processes can also create new niches for new plant species (Bobbink et al., 2010; Yang et al., 2011; Cai et al., 2023). Thus, soil acidification and eutrophication may not necessarily result in plant species loss. An increase in the abundance of C_3_ plant species might be related to their high potential to acquire and utilize essential resources (Zhang et al., 2021b), functional types (Harpole et al., 2007; Reich et al., 2001; Cai et al., 2023), and increased ammonium and ammonia availability (Bobbink et al., 2010).

Additionally, steppes had asymmetric sensitivity to experimental manipulations. At the community level, plant species richness was insensitive to N (Yu and He, 2019) whereas steppe composition was sensitive to N. These results are contrary to previous studies (Stevens et al., 2010; Harpole et al., 2016). At the ecosystem level, aboveground biomass was more sensitive to water and N manipulations than belowground biomass. In nature, belowground biomass is determined by root production and turnover (Majdi and Andersson, 2005; Leppälammi-Kujansuu et al., 2014; Keller et al., 2023). N addition enhanced aboveground production, which could be attributed to faster growth in favorable conditions (Ren et al., 2015; Yu and He, 2017; Peng et al., 2024) and the fact that the positive effects of N on aboveground production were stronger than the negative effects of plant species richness.

In summary, the potential of simulated N deposition to improve grasslands was not amplified by increasing rainfall. As opposed to our currently thought, high rainfall increase might weaken the efficacy of high N deposition on steppes. The complex interplay between rainfall changes and N deposition plays a vital role in determining steppe trajectories during postgrazing succession. Recognizing such interactions among multiple global change drivers is essential for predicting and managing grassland ecosystems.

## Data Availability

The original contributions presented in the study are included in the article/**Supplementary Material**. Further inquiries can be directed to the corresponding author.
