# Supplementary material for: Rainfall and nitrogen addition have no synergistic effects on steppe composition and production in postgrazing succession
Source: Front Plant Sci. 2025 Sep 5;16:1635593. doi: 10.3389/fpls.2025.1635593 (PMC12447802; doi:10.3389/fpls.2025.1635593)

**Supplementary Materials**

**Table** S1 PLFA biomarkers used to indicate microbial groups.

| Microbial group | Phospholipids fatty acid signatures |
| --- | --- |
| Bacteria | 12:0, 12:0a, 12:0i, 12:0 2OH, 12:0 3OH, 13:0i, 14:0, 14:0i, 15:0, 15:0a, 15:0i, 15:0i 3OH, 16:0a, 16:0i, 16:1w7c, 16:1w9c, 16:1 2OH, 17:0, 17:0a, 17:0i; 17:0cy, 17:0 10ME, 17:1w8c, 18:0 10ME, 18:1 w7c, 19:0cy |
| Actinomycetes | 17:0 10ME, 18:0 10ME |
| Arbuscular mycorrhizal fungi (AMF) | 16:1w5c |
| Fungi | 16:1w5c, 18:1w9c, 18:3w6c, |
| Protozoa | 20:4 w6,9,12,15c |

**References**

Chung, H., Zak, D.R., Reich, P.B., and Ellsworth, D.S. (2007). Plant species richness, elevated CO_2_, and atmospheric nitrogen deposition alter soil microbial community composition and function. *Glob. Change Biol.* 13, 980–989.

Kourtev, P.S., Ehrenfeld, J.G., and Häggblom, M. (2002). Exotic plant species alter the microbial community structure and function in the soil. *Ecology* 83, 3152–3166.

Rinnan, R., Michelsen, A., Bååth, E., and Jonasson, S. (2007). Fifteen years of climate chagne manipulations alter soil microbial communities in a subarctic heath ecosystem. *Glob. Change Biol.* 13, 28–39.

Zak, D.R., and Kling, G.W. (2006). Microbial community composition and function across an arctic tundra landscape. *Ecology* 87, 1–67.

**TABLE S2** The effects of rainfall increase, N addition, and their interactions on soil properties. Values of *P* ≤ 0.05 are in bold

|  |  |  | Rainfall increase (R) | |  | Nitrogen addition (N) | |  | R × N | |
| --- | --- | --- | --- | --- | --- | --- | --- | --- | --- | --- |
| Variables | |  | *χ^2^* | *P* |  | *χ^2^* | *P* |  | *χ^2^* | *P* |
|  | Bacteria |  | 3.610 | 0.165 |  | 1.669 | 0.434 |  | 3.115 | 0.539 |
|  | AMF |  | **7.281** | **0.026** |  | **39.30** | **<0.001** |  | **9.547** | **0.049** |
|  | Actinomycetes |  | 5.215 | 0.074 |  | 1.297 | 0.523 |  | 3.079 | 0.545 |
|  | Fungi |  | **7.583** | **0.022** |  | **15.32** | **<0.001** |  | 8.287 | 0.081 |
|  | Fungi/Bacteria ratio |  | 2.699 | 0.259 |  | **43.90** | **<0.001** |  | 8.759 | 0.067 |
|  | Protozoa |  | 1.809 | 0.405 |  | 0.148 | 0.929 |  | 7.768 | 0.101 |
|  | NH_4_^+^-N |  | 2.209 | 0.331 |  | **37.54** | **<0.001** |  | 1.198 | 0.878 |
|  | NO_3_^-^-N |  | 3.812 | 0.149 |  | **23.32** | **<0.001** |  | 2.249 | 0.690 |
|  | pH |  | 1.744 | 0.418 |  | **87.88** | **<0.001** |  | 5.362 | 0.252 |
|  | Available P |  | 4.402 | 0.111 |  | 4.612 | 0.099 |  | 5.443 | 0.244 |
|  | Total P |  | 3.938 | 0.140 |  | 1.750 | 0.417 |  | 5.938 | 0.204 |
|  | Total N |  | 3.865 | 0.145 |  | 0.607 | 0.738 |  | 6.680 | 0.154 |
|  | Total C |  | 4.606 | 0.099 |  | 3.786 | 0.151 |  | **9.583** | **0.048** |

**FIGURE LEGENDS**

**FIGURE S1** Effects of increased rainfall and N addition on soil microbes. AMF, arbuscular mycorrhizal fungi; Fungi/Bacteria ratio, ratio of fungi to bacteria. R0: no rainfall addition; R14: a 14% increase in rainfall amount over the ambient rainfall; R28: a 28% increase in rainfall amount over the ambient rainfall; N0: no N addition; N5: an addition of 5 g N m^-2^ yr^-1^; N10: an addition of 10 g N m^-2^ yr^-1^. The data are expressed as means + 1 SE.

**FIGURE S2** Effects of increased rainfall and N addition on soil nutrients. The data are expressed as means + 1 SE. Treatment abbreviations are from Figure S1.

**FIGURE S3** Effects of increased rainfall and N addition on soil pH. The data are expressed as means + 1 SE. Treatment abbreviations are from Figure S1.

**FIGURE S1**

**
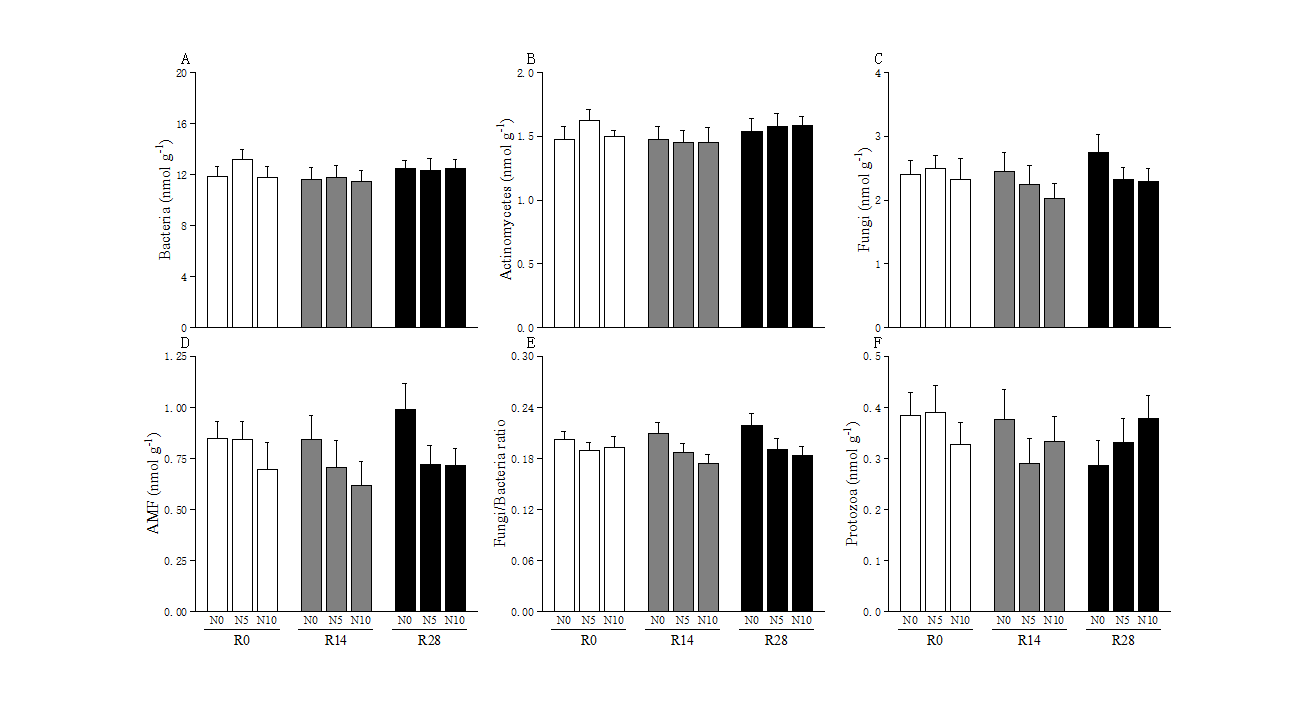
**

**FIGURE S2**


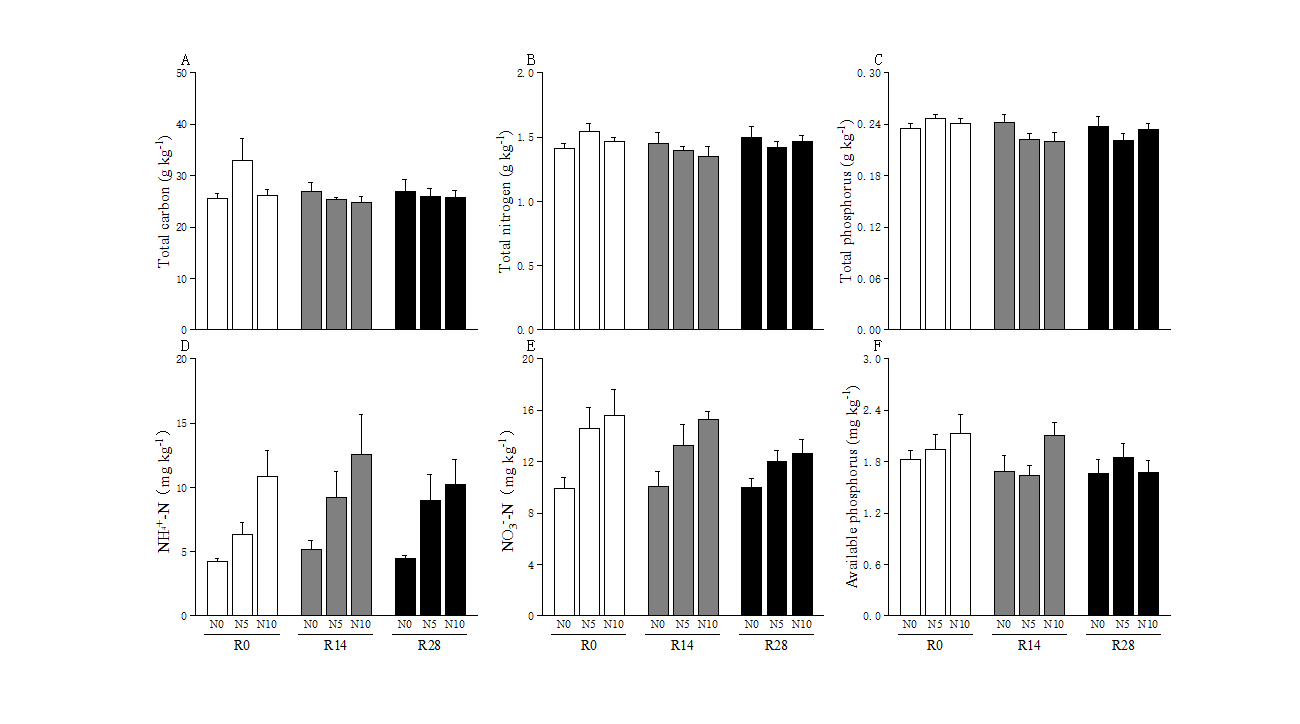


**FIGURE S3**


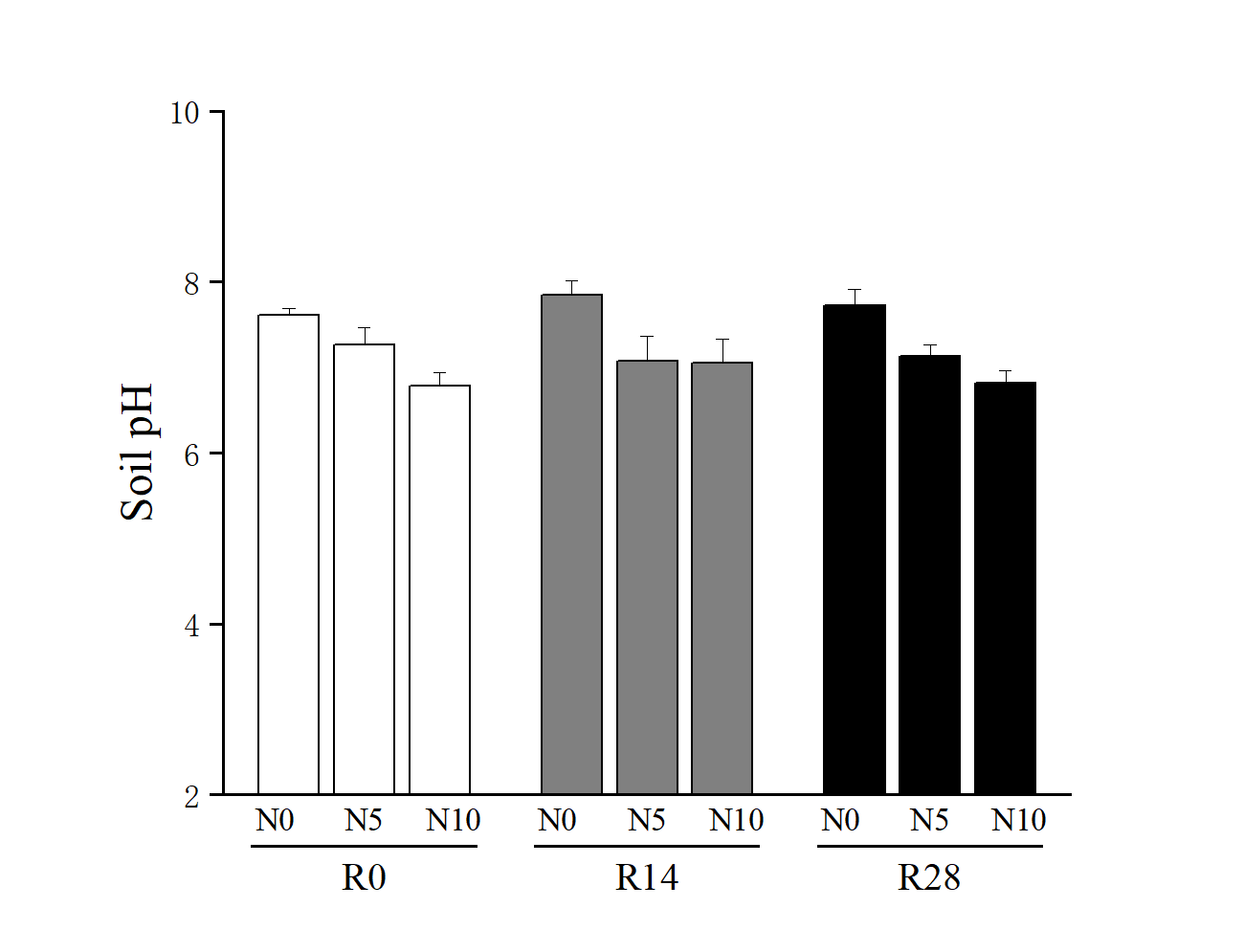

Supplement: Supplementary file 1 [file Table1.doc]
